# Effects of a commercially available branched-chain amino acid-alanine-carbohydrate-based sports supplement on perceived exertion and performance in high intensity endurance cycling tests

**DOI:** 10.1186/s12970-020-0337-0

**Published:** 2020-01-20

**Authors:** Marco Gervasi, Davide Sisti, Stefano Amatori, Sabrina Donati Zeppa, Giosuè Annibalini, Giovanni Piccoli, Luciana Vallorani, Piero Benelli, Marco B. L. Rocchi, Elena Barbieri, Anna R. Calavalle, Deborah Agostini, Carmela Fimognari, Vilberto Stocchi, Piero Sestili

**Affiliations:** 1 Department of Biomolecular Sciences, University of Urbino Carlo Bo, Urbino, Italy; 2 Interuniversity Institute of Myology, Perugia, Italy; 3 Department for Life Quality Studies, Alma Mater Studiorum, University of Bologna, Rimini, Italy

**Keywords:** Supplements, Branched-chain amino acids (BCAA), Central fatigue, Rating of perceived exertion (RPE), Performance, Endurance exercise, Training impulse (TRIMP), Creatine kinase (CK)

## Abstract

**Background:**

Sports nutritional supplements containing branched-chain amino acids (BCAA) have been widely reported to improve psychological and biological aspects connected to central fatigue and performance in endurance exercise, although the topic is still open to debate. The aim of the present study was to determine whether the intake of a commercially available BCAA-based supplement, taken according to the manufacturer’s recommendations, could affect the rating of perceived exertion (RPE) and performance indexes at the beginning (1d) and end of a 9-week (9w) scheduled high intensity interval training program, with an experimental approach integrating the determination of psychometric, performance, metabolic and blood biochemical parameters.

**Methods:**

This was a randomized double-blind placebo-controlled study. Thirty-two untrained, healthy young adults (20 males and 12 female) were enrolled. A high-intensity endurance cycling (HIEC) test was used to induce fatigue in the participants: HIEC consisted in ten 90 s sprints interspersed by ten 3 min recovery phases and followed by a final step time to exhaustion was used. In parallel with RPE, haematological values (creatine kinase, alanine, BCAA, tryptophan, ammonia and glucose levels), and performance indexes (maximal oxygen consumption - VO_2max_, power associated with lactate thresholds - W_LT1_, W_LT2_ and time to exhaustion - TTE) were assessed. All subject took the supplement (13.2 g of carbohydrates; 3.2 g of BCAA and 1.6 g of L-alanine per dose) or placebo before each test and training session. Dietary habits and training load were monitored during the entire training period.

**Results:**

The administration of the supplement (SU) at 1d reduced RPE by 9% during the recovery phase, as compared to the placebo (PL); at 9w the RPE scores were reduced by 13 and 21% during the sprint and recovery phase, respectively; at 9w, prolonged supplement intake also improved TTE and TRIMP. SU intake invariably promoted a rapid increase (within 1 h) of BCAA serum blood levels and prevented the post-HIEC tryptophan: BCAA ratio increase found in the PL group, at both 1d and 9w. There was no difference in dietary habits between groups and those habits did not change over time; no difference in glycemia was found between SU and PL. VO_2max_, W_LT1_ and W_LT2_ values improved over time, but were unaffected by supplement intake.

**Conclusions:**

On the whole, these results suggest that i) the intake of the BCAA-based commercially available supplement used in this study reduces RPE as a likely consequence of an improvement in the serum tryptophan: BCAA ratio; ii) over time, reduced RPE allows subjects to sustain higher workloads, leading to increased TRIMP and TTE.

## Background

Amino acids are thought to enhance athletic performance in several ways, for example modifying fuel utilization during exercise and preventing mental fatigue and overtraining [1]. A recent (2017) position stand of the International Society of Sports Nutrition [2] states that the three branched-chain amino acids (BCAA), leucine, isoleucine, and valine are unique among the essential amino acids for their roles in protein metabolism, neural function, blood glucose and insulin regulation. It has been suggested that the Recommended Dietary Allowance (RDA) for sedentary individuals (considering that BCAAs occur in nature in a 2:1:1 ratio, leucine: isoleucine: valine) should be 45 mg/kg/day for leucine and 22.5 mg/kg/day for both isoleucine and valine; this RDA is even higher for active individuals [3]. Moreover the European Food Safety Authority indicated an amount recommendation between 3 g and 12 g per day (higher dose may lead to ammonia build-up) [4]. Supplementation with BCAA has been proposed as a possible strategy to limit the development of central fatigue [5], in particular, in endurance events [1]. Central fatigue, which pertains to the central nervous system (CNS), is a complex phenomenon arising under conditions of low energy availability [6, 7], ammonia accumulation in blood and tissues [8], and changes in neurotransmitter synthesis - in particular, an increase in serotonin and a decrease in dopamine - which causes a state of increasing tiredness during exhaustive exercise [9]. The presence of elevated cerebral serotonin levels observed in rats under fatigue [10], is the basis of a well-accepted theory to account for the onset/increase of central fatigue in humans as well. Indeed, during prolonged sustained exercise, an increased brain uptake of the serotonin precursor Tryptophan (Trp) has been observed in humans [11, 12]. This theory has recently been bolstered by Kavanagh et al. [13], whose study based on paroxetine administration in humans demonstrated the influence of serotonin availability in increasing central fatigue under prolonged maximal contractions. The ability of BCAA to compete with Trp in crossing the blood brain barrier led us to hypothesize that BCAA supplementation could reduce cerebral serotonin synthesis, thus preventing/delaying the onset of central fatigue during prolonged exercise [14, 15].

In addition to BCAA, other amino acids reputed to play a role in maintaining performance during endurance exercise are often included in sports supplements. Among these, L-alanine (Ala, another component of the product tested in the present study) is thought to support performance through several mechanisms [16], including the prevention of an exercise-induced decrease in many gluconeogenic amino acids and hence a metabolic profile that enhances performance [17]. Ala is consumed in quantities of 3 to 4 g/day on average in a typical diet; however, no studies have assessed the long-term effects of its supplementation in humans alone or combined with BCAA [18].

Carbohydrates (CHO) also play an important role in supplementation in the course of endurance events, increasing and/or maintaining energetic substrate availability [19], preventing and/or delaying hypoglycemia and its deleterious effects on brain functions and cognitive performance, and promoting direct anti-fatigue brain responses through the activation of sweet taste oral receptors [5].

In light of these findings, researchers have turned their attention to the study and development of supplements containing BCAA alone or combined with specific substances (such as CHO), assessing the efficacy of their association [15, 20]. Several recent investigations have shown BCAA supplementation to positively affect prolonged exercise under specific conditions. In particular, BCAA were shown to positively impact the rating of perceived exertion (RPE) [14] and performance [21, 22]. However, due to the great heterogeneity of the experimental protocols and formulations used, the results of these studies are not always unequivocal; hence, the actual efficacy of BCAA – used alone or combined with other components - remains a much debated issue [15, 23, 24].

This uncertainty may generate confusion and/or false expectations regarding the efficacy of these sport supplements. To shed light on this issue, it is important to perform highly controlled and randomized studies as well as to develop and validate specific and reliable test procedures capable of determining the actual efficacy of supplements intended for use in sports after both short and long term intakes [25]. To this end, a recent study [26] validated a variable high intensity protocol followed by a time to exhaustion (TTE) endurance capacity test (namely high intensity endurance cycling test, HIEC) as a reliable and sensitive method to assess both performance and fatigue, providing a stable platform for the comparative analysis of the effects of different nutritional interventions. HIEC can be performed either at the beginning or at the end of training periods and protocols. In the present study, we applied HIEC to a 9-week program based on High Intensity Interval Training (HIIT), a widely used protocol to improve specific variables of endurance performance [27, 28]. It is worth noting that, to date, to the best of our knowledge, no study has tested the effects of the consumption of a commercially available and established BCAA-alanine-CHO based supplement on HIEC over a medium-long endurance training period.

The first aim of this randomized double-blind placebo-controlled study was to determine whether, the single or prolonged intake of a commercial BCAA, Ala and CHO formula (Friliver® Performance, FP, Dompè Farmaceutici Spa), taken according to the manufacturer’s recommendations, affects RPE [29], performance indexes (maximal oxygen consumption, VO_2max_; peak power, W_peak_; power at lactate thresholds, W_LT1_ and W_LT2_; and TTE) and relevant serum blood markers (creatine kinase - CK, Ala, BCAA, Trp:BCAA ratio and glycemia) in young adults, at the beginning (1d) and at the end (9w) of a 9-week indoor cycling HIIT [26]. The second aim was to verify whether a prolonged supplementation may help participants to comply with the required training load during a 9w HIIT program with progressively increasing volume.

## Methods

### Participants

Thirty-two healthy university students (20 males: age 22 ± 1.7 years, height 175.5 ± 6.5 cm, weight 68.2 ± 10.9 kg, BMI 22 ± 2.7 kg/m^2^; 12 females: age 21 ± 0.9 years, height 159.5 ± 4.8 cm, weight 52.5 ± 5.3 kg, BMI 21 ± 1.2 kg/m^2^) were recruited. The exclusion criteria were: major cardiovascular disease risks, musculoskeletal injuries, upper respiratory infection, smoking and consumption of any medicine or protein/amino acid supplement in the past 3 months. All participants, assessed with a specific questionnaire, performed no more than one 60 min leisure walking or jogging session per week in the 3 months preceding the start of the study; their VO_2max_ values at baseline were in line with- and thus confirmed - their low level of training (see Table 2). The participants were advised to maintain their dietary routine, and to abstain from using additional dietary supplements during the study period. They were also instructed to refrain from all training activities except for the sessions included in the experimental design. Subjects were asked to refrain from the consumption of alcohol, hypnosedative drugs and beverages containing caffeine on the 2 days prior to the trial. Following a medical health-screening, all participants provided written informed consent to participate in the study, which was approved by the Ethics Committee of the University of Urbino Carlo Bo, Italy (02/2017, date of approval July 10, 2017) and was conducted in accordance with the Declaration of Helsinki for research with human volunteers (1975).

### Study design

This was a randomized double-blind placebo-controlled trial (2/2017, conducted according to Good Clinical Practice). In order to ensure balance, randomization for permuted blocks (*n* = 4) was used. Stratification was used to ensure equal allocation by gender to each experimental condition. Study design was structured as follows: metabolic/performance (VO_2max_, W_peak_, W_LT1_, W_LT2_ and TTE), biochemical (BCAA, Ala, Trp, CK serum and glucose blood levels) and RPE data were acquired before (1d) and after (9w) the incremental training period.

#### Supplement and supplementation regimen

FP (Dompè Farmaceutici Spa, Milan, Italy, see Table 1 for the formulation) was taken 1 h before HIEC and each training session according to the manufacturer’s recommendations. BCAA and Ala content per single dose is within the range recommended by European Food Safety Authority and comparable to the dosage used in other studies [4, 18, 30]. The PL group ingested a non-caloric placebo that was identical in packaging, appearance and taste to the actual supplement. FP and PL were dissolved in 500 ml of still water and ingested before each training session; neither FP nor PL was taken on rest days. Over the entire study period, the SU group received an average daily dose (total amount of each amino acid in FP/duration in days of the study) of 0.91 g leucine, 0.46 g valine, 0.46 g isoleucine and 0.91 g alanine. Importantly, as verified by the qualified medical specialist (P.B.), none of the participants experienced any side effects or adverse events as a result of the FP or placebo ingestion.

**Table 1:** Composition of Friliver Performance®

	Per dose	Per 100 g
Energy	71 kcal (304 kJ)	355 kcal (1520 kJ)
Total Carbohydrate	13.2 g	66 g
Sucrose	11.6 g	58 g
Polyalcohol	1.6 g	8 g
L-Leucine	1.6 g	8 g
L-Alanine	1.6 g	8 g
L-Valine	0.8 g	4 g
L-Isoleucine	0.8 g	4 g
Citric acid	1.06 g	5 g
Orange flavor	0.8 g	4 g

#### Incremental test

Prior (3 days before) to the pre- and post- training experimental sessions, each subject performed an incremental test to assess individual VO_2max_, W_peak_, W_LT1_ and W_LT2_. Male subjects started cycling on an electronically-braked ergometer (SRM Italia, Lucca, Italy) at 75 W, and power output was increased by 25 W every 3 min, whereas female subjects started at 50 W, and power output was increased by 20 W every 3 min. All subjects continued increasing power output until volitional exhaustion or cadence dropped below 60 rpm [31, 32]. In the absence of specific literature, intervals were set at 3 min, which represents an appropriate compromise with previous data on incremental exercise test design [33, 34]. Oxygen consumption was monitored breath-by-breath using a Cosmed K4b2 metabolimeter, (COSMED, Rome, Italy) and values of heart rate (HR) (assessed with a Polar RS-800 HR monitor, POLAR, Kempele, Finland) were recorded continuously; VO_2max_ was calculated according to Robergs et al. [35]; blood lactate was measured before starting the test and in the 15 s before the end of each stage using a Lactate-Pro (portable blood lactate meter, Arkray, Kyoto, Japan) on micro blood samples drawn from the tip of the index finger. As already experimented in a previous study [36], and according to Seiler et al. [37] lactate blood levels ([La]) were used to calculate the power at lactate thresholds of [La] 2.0 mmol /L (W_LT1_) and [La] 4.0 mmol/L (W_LT2_) and then identify the three HR training intensity zones. The scheme was: zone 1: [La] < 2.0 mmol/L; zone 2: 2.0 < [La] < 4.0 mmol/L; zone 3: [La] > 4.0 mmol/L [36, 38]. W_peak_ was calculated as follows: *W*_*peak*_ = *Wf + [(t/D x P)]*, where *Wf* is the power output during the last completed stage, *t* is the duration of the last uncompleted stage, *D* is the duration of each stage in seconds (=180 s) and *P* is the incremental increase in power output with every stage [39].

#### Rating of perceived exertion

RPE was determined with the 0–10 OMNI-cycle scale, which combines mode-specific pictorial illustrations with a numerical rating format, using a procedure described in the literature [40, 41]. A standard definition of perceived exertion (“the subjective intensity of effort, strain, discomfort, and fatigue that was felt during exercise”) and instructional sets for the OMNI scale were read to the subjects immediately before the exercise test [41]. The initial exercise anchoring procedure was illustrated and performed during the incremental test (see “*Incremental Test”* section). Participants were asked to point to their RPE on the OMNI-cycle scale, which was in full view at all times during testing.

#### HIEC test

The HIEC test was performed on a power meter-provided bike “Technogym Group Cycle™ Connect” (Technogym S.p.A., Cesena, Italy). To preliminarily calculate the individual workload, a modified O’Hara protocol [42] based on W_peak_ was adopted. Following a warm up stage (four 5 min continuous progressive increments at a workload corresponding to 50, 60, 65 and 70% W_peak_), participants performed ten 90 s sprints (SPR) at 90% W_peak_, separated by 180 s recovery (REC) at 55% W_peak_. The subjects capable of completing all the 10 SPR recovered for an additional 3 min at 55% W_peak_, and then performed a final TTE step at 90% W_peak_. Exhaustion was defined as the inability to maintain power output within 5 W of the target output for 15 s despite verbal encouragement; no feedback on elapsed time was provided. TTE was taken as a performance marker. Subjects were asked to maintain the same predefined cadence throughout the HIEC regardless of the power output variations (from 90 to 55% W_peak_) introduced by the operator at each REC/SPR change [26]. Subjects were asked to provide their RPE 10 s before the end of each of the warm up, SPR and REC steps [29].

Immediately after the incremental test,. 3 days before the experimental session, the subjects performed a shortened version of the HIEC test so that they would be familiar with the test [26].

### Design of the 1d and 9w experimental training sessions

**Fig. 1:**
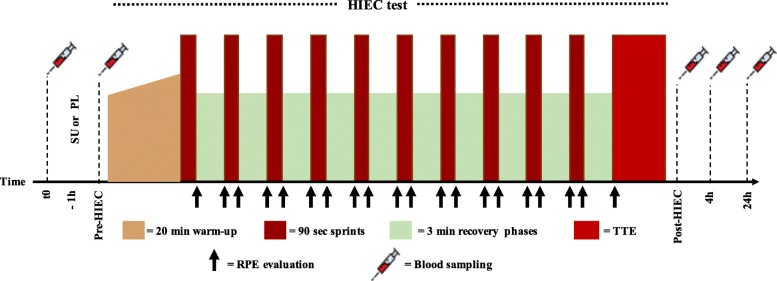
Design of the experimental sessions at 1d and 9w. The experimental sessions were performed in the morning. 1 h after breakfast, participants had their first blood draw immediately before the consumption of SU or PL; after another 1-h interval, a second blood sampling was performed immediately before the beginning of the HIEC (Pre-HIEC). In the course of the HIEC, RPE was repeatedly evaluated as indicated by the arrows. Further blood samples were collected immediately, at 4 and 24 h after the completion of the HIEC

The 32 subjects were divided in 4 groups of 8, and they performed the HIEC test on two consecutive days (2 groups per day). On the experimental day, subjects in the first group arrived at the laboratory at 06.00 AM, 2 h before the test, in a fasted state. The second group of the day arrived 2 h later in a fasted state. All subjects had a standardized breakfast consisting of 400 ml of fruit juice and servings of jam tart adjusted according to gender caloric needs (90 g for females and 135 g for males; total breakfast calories: 612–794 kcal, 119.6–150.6 g CHO, 6–8.4 g Protein, 11.4–16.9 g Fat). Breakfast total calories represented about 30% of the Total Energy Intake, calculated using the FAO equation, with a coefficient of 1.55 (male) and 1.56 (female) to take into account the Physical Activity Level (light activity) [43]. The design of the experimental session is shown in Fig. 1.

#### Blood sampling and analysis

Venous blood samples (5 ml) were obtained from the antecubital vein and collected in BD Vacutainer® SST™ blood collection tubes (BD diagnostic preanalytical systems, Milan, Italy) 1 h after breakfast (immediately before FP or PL ingestion) (T0), 1 h after ingestion (immediately before exercise) (pre-HIEC), immediately post exercise (post-HIEC), after 4 h and 24 h. Serum was obtained from clotted blood by centrifugation at 1000 g at 4 °C for 15 min and stored at − 80 °C for later analyses. Serum CK activity was measured at pre-HIEC, post-HIEC, 4 h and 24 h by a standardized commercially available colorimetric enzymatic assay (BioVision, Vinci-Biochem, Italy). Ammonia levels at T0, pre and post-HIEC were measured using a commercially available assay (Sigma Ammonia Assay Kit, Sigma-Aldrich, USA). The serum blood levels of BCAA, Ala, total and free Trp were determined at T0, pre and post-HIEC, by HPLC according to Stocchi et al. [44]. The intra and inter-assay confidence interval for CK kit is ≤10.0% for both values; for ammonia determination assay kit 4–7 and 5–8% values, respectively.

#### Glycemia assessment

Blood glucose was measured by a portable glucometer (MyStar Extra, Sanofi) [45] at the following times: T0 in the fasted state; immediately and 30 min after breakfast; before the intake of FP or PL (. 1 h after the standardized breakfast); 30 min after intake of SU or PL; and immediately before and after the HIEC test.

### Training protocol

**Fig. 2:**
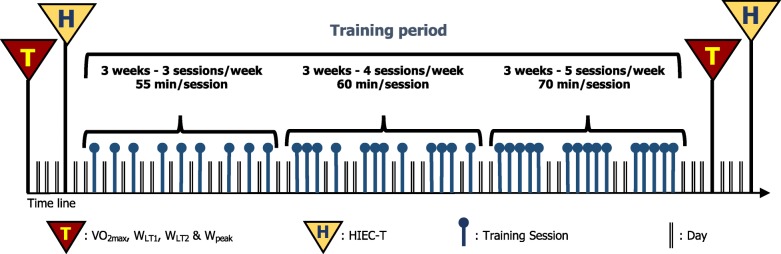
Structure of training period: nine weeks divided into three mesocycles (three weeks each). The frequency and the duration of the sessions are also indicated. Key: VO_2max_, maximal oxygen consumption; W_LT1_ and W_LT2_, power at lactate thresholds; W_peak_, peak power; HIEC-T, high intensity endurance cycling test

Thirty-six indoor cycling training sessions were performed over a 9w period (see Fig. 2). The training sessions were divided into three mesocycles, as follows:

First: three 53.1 ± 1.3 -min sessions per week over a 3-week period;

Second: four 59.1 ± 1.2 -min sessions per week over a 3-week period;

Third: five 68.2 ± 1.4 -min sessions per week over a 3-week period.

The 32 subjects were divided in two groups of 16 and trained by two expert instructors with the aim of following the same training program. Each session was choreographed based on conventional principles (. warm-up, systematic high intensity interval exercise, and cool-down) widely used in the indoor cycling community [46]. The training program of each session was designed following the same intensity distribution, based on a polarized model, with around 70% of the session time spent in zone 1, 10% spent in zone 2 and 20% spent in zone 3 (see “*Incremental Test”* section for zone determination), according to Seiler and Kjerland [38]. During the training sessions, the HR of each subject (instructor included) was monitored and recorded using a Polar Team Pack 2 (POLAR, Kempele, Finland). HR values were projected onto the wall, as percentage of maximal HR (% HR_max_), and the subjects were asked to maintain the same intensity as the instructor.

One hour before each training session, the subjects of the SU group ingested a single dose of FP, while the subjects of the PL group ingested the placebo.

### Training load analysis

Lucia’s TRIMP [47] was used to calculate the Training Load for each session. The concept of Lucia’s TRIMP integrates total volume, on the one hand, and total intensity relative to the intensity zones, on the other. Briefly, the score for each zone is calculated by multiplying the accumulated duration in the zone by a multiplier for that particular zone (e.g. 1 min in zone 1 is given a score of 1 TRIMP (1 X 1), 1 min in Zone 2 is given a score of 2 TRIMP (1 X 2), and 1 min in Zone 3 is given a score of 3 TRIMP (1 X 3); the total TRIMP score is then obtained by summing the results of the three zones [47]. Finally, the mean TRIMP scores of each mesocycle performed by the SU and PL groups were compared.

### Diet & Diet Tracking

During the entire training period, subjects’ nutrition was monitored daily (by call interviews, always carried out after dinner) and data were collected and processed using MètaDieta software (METEDA S.r.l., San Benedetto del Tronto, Italy); macronutrients and total energy intake for experimental and control groups were finally compared in order to exclude differences in nutritional habits.

### Statistical analysis

Descriptive statistics were performed using means and standard deviations. Homogeneity between groups was tested using the unpaired *t*- test. Daily protein, fat, carbohydrate and total caloric intake were compared between groups; the t-test and Cohen’s effect size (ES) [48] were used to quantify differences. For Cohen’s d, an ES of 0.2–0.3 was considered a “small” effect, around 0.5, a “medium” effect, and 0.8 to infinity, a “large” effect [48]. The time series of the RPE analysis were performed using the HIEC test values for each of the four conditions (1d SPR, 1d REC, 9w SPR, 9w REC) comparing the PL and SU groups. For each of the four conditions, differences between slopes and intercept (SU vs PL) were tested using the statistical approach according to Dupont and Plummer [49]. Furthermore, in all experimental conditions, SPR RPE values were plotted against delta RPE (SPR - REC) in order to verify the degree of recovery in REC steps. Two-way ANOVA with interaction was used in 1d and 9w conditions to test the association among group partnership (SU vs PL) and gender, used as predictive binary factors, and TTE, used as a dependent variable. Partial eta-squared (*η*^*2*^*)* values were used as ES. VO_2max_, W_peak_, W_LT1_ and W_LT2_ were compared between 1d and 9w training using ANOVA for repeated measures, using groups as a between categorical predictive factor. According to Cohen (1988) [48], an *η*^*2*^ ranging from 0.02 to 0.13 was considered a “small” effect, from 0.13 to 0.26 a “medium” effect, and higher than 0.26 a “large” effect. TRIMPs were compared between groups as the mean of sessions of each mesocycle. TRIMP comparison was performed using a two-way ANOVA with interaction, followed by the LSD post-hoc test. Similarly, glucose levels were compared at different measurement times. Finally, CK, total BCAA, alanine, total Trp, free Trp, Trp:BCAA and ammonia levels were compared using a two-way ANOVA for repeated measures. For CK levels, time (pre-HIEC, post-HIEC, 4 h and 24 h CK levels) was within factor, and group membership (SU vs PL) was between factor. Contrast analysis for differences between two consecutive measures (post-HIEC vs pre-HIEC; 4 h vs post-HIEC; 24 h vs 4 h) versus the group was performed. CK levels were also plotted versus TRIMP values during HIEC, and correlation analysis was performed. For total BCAA, alanine, total Trp, free Trp and Trp:BCAA, time was within factor (t0, pre-HIEC and post-HIEC) and group membership was between factor. All statistical analyses were performed using Excel or SPSS 20.0; the significance threshold was fixed at 0.05.

## Results

### Baseline anthropometric, metabolic and biomechanical variables

Anthropometric, metabolic and biomechanical variables of participants were assessed before the beginning of the experimental session as reported in Table 2. No differences were found between the two groups in the tested parameters.

**Table 2:** Anthropometric, metabolic and biomechanical variables of the participants at baseline; Mean, standard deviations and *p* values for group are reported

	Supplemented Group (*n* = 16)		Placebo Group (*n* = 16)		Group (*p*)
Participants	Males = 10	Females = 6	Males = 10	Females = 6	
Age (yr)	22.1 ± 2.2	20.6 ± 1.0	21.0 ± 1.0	20.5 ± 0.7	0.322
Height (cm)	173.9 ± 6.0	157.2 ± 4.4	177.1 ± 6.8	161.8 ± 4.3	0.072
Weight (kg)	69.2 ± 12.7	50.3 ± 4.8	67.2 ± 6.9	54.6 ± 5.2	0.726
BMI (kg/m^2^)	22.8 ± 3.4	20.3 ± 1.2	21.4 ± 1.6	20.8 ± 1.4	0.584
HR_max_ (bpm)	197.3 ± 8.2	197.5 ± 5.0	199.0 ± 7.8	199.7 ± 4.7	0.458
VO_2max_ (ml/kg/min)	42.6 ± 10.4	35.0 ± 8.5	43.9 ± 4.5	28.1 ± 3.1	0.315
W_peak_ (watt)	212.5 ± 33.9	146.7 ± 23.4	230.0 ± 28.4	133.3 ± 23.4	0.844
W_LT1_ (watt)	76.1 ± 11.3	57.7 ± 23.8	73.9 ± 22.3	40.8 ± 14.0	0.312
W_LT2_ (watt)	127.1 ± 23.4	88.2 ± 23.5	138.8 ± 27.6	74.9 ± 16.6	0.928

### Diet monitoring

Daily caloric intake over the study period was virtually identical for both groups: 1944 ± 876 kcal in the SU group vs 2043 ± 947 in the PL group, with no significant difference (t test; *p* > 0.05); ES showed a negligible effect (ES = 0.07).

Daily CHO, fat and protein intakes, supplemented vs placebo group were 49.1% vs 51.1%; 33.4% vs 32.4%; 17.4% vs 16.9%, respectively. No differences in specific macronutrient intake were found between groups (*t* test; *p* > 0.05); a very small, negligible effect size was observed for carbohydrates, fats and proteins: 0.12, 0.07 and 0.06, respectively.

### VO_2max_, W_peak_ and power at lactate thresholds at 1d and 9w

All these variables, namely VO_2max_, W_peak_, W_LT1_ and W_LT2_, were significantly different in pre vs post 9w training as shown in Table 3. For all variables, *p* values were < 0.001. Results indicate that all post training values were significantly greater than pre-training ones, with partial *η*^*2*^ > 0.484 (large effect). The effect of SU intake was not significant (*p* > 0.05) for all dependent variables.

**Table 3:** VO_2max_, W_peak_, W_LT1_ and W_LT2_*, in SU and PL groups at 1d and 9w

	Supplemented Group			Placebo Group		
	1d	9w	Δ%	1d	9w	Δ%
VO_2max_	39.73 ± 10.18	44.58 ± 6.67	+ 12%	37.95 ± 8.82	42.93 ± 5.54	+ 13%
W_peak_	187.81 ± 44.20	231.56 ± 48.91	+ 23%	193.75 ± 54.79	239.06 ± 56.01	+ 23%
W_LT1_	69.21 ± 18.69	103.64 ± 32.95	+ 50%	64.83 ± 27.12	91.03 ± 26.56	+ 40%
W_LT2_	112.50 ± 29.85	155.56 ± 35.34	+ 38%	114.83 ± 39.59	156.51 ± 40.66	+ 36%

* VO_2max_: maximal oxygen consumption (ml*kg^−1^*min^− 1^); W_peak_: power peak (watt); W_LT_: power at Lactate Threshold (watt); Δ%: percentage difference in average values

### Perceived exertion during HIEC test

**Fig. 3:**
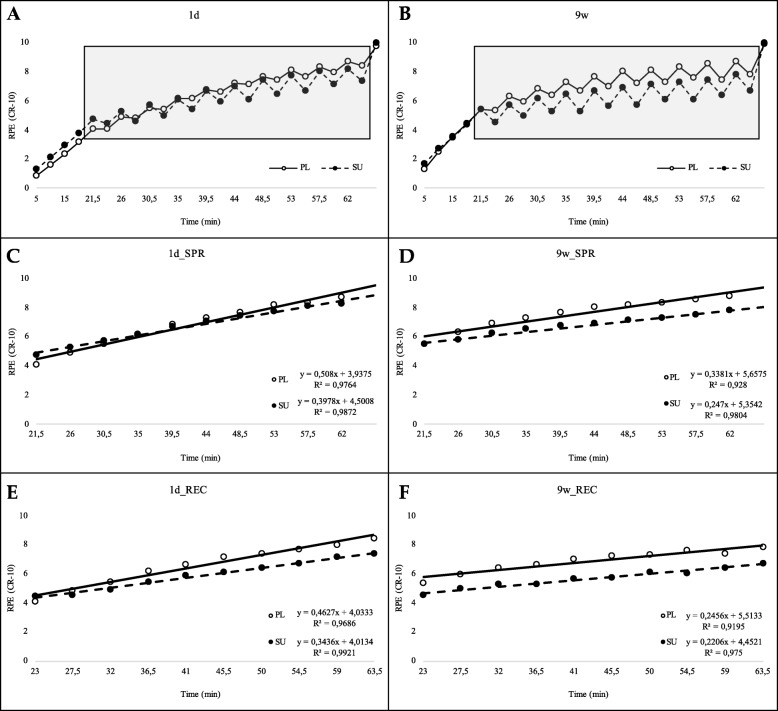
Perceived exertion rate (RPE) values versus session time; **a**-**b** whole RPE time series; **c**-**d** RPE values in sprint (SPR) steps at pre-training (1d) and post training (9w) stages, respectively; **e**-**f** RPE values in REC steps at 1d and 9w, respectively. Closed circles refer to SU and open circles to the PL group

RPE values, measured during the 20 min warm up of the HIEC tests increased progressively, showing a very similar trend in the PL and SU groups in both 1d and 9w periods (Fig. 3a and b, respectively). During the 10 SPR, each of them followed by a REC step, RPE showed an upward trend characterized by a sawtooth pattern in all the conditions tested. As expected, the RPE values reached the maximum at the end of the TTE step (11 points on OMNI cycle scale). Hence, only RPE values starting from 20 min (. the end of warm up) to 65 min (. prior to TTE phase) were considered for further analyses (data highlighted in grey box).

#### Perceived exertion at 1d (pre training HIEC test)

The linear regression equation of the curve built on SPR steps’ data in PL group was *RPE*_*HIEC*_ *= 0.508 time+ 3.937* (*r*^2^ = 0.98) vs *RPE*_*HIEC*_ *= 0.398 time+ 4.501* (*r*^2^ = 0.99) in the SU group. Intercepts (*p* = 0.163) and slopes (*p* = 0.086) were not significantly different. The linear regression equation of REC steps’ data in the PL group was *RPE*_*HIEC*_ *= 0.463 time+ 4.033* (*r*^2^ = 0.97) vs *RPE*_*HIEC*_ *= 0.344 time+ 4.013* (*r*^2^ = 0.99) in the SU group. Intercepts were not significantly different (*p* = 0.742), whereas, interestingly, slopes were (*p* = 0.001). This would imply that in REC steps, the SU group showed a lower RPE (Fig. 3c e 3E).

#### Perceived exertion at 9w (post training HIEC test)

The linear regression equation of SPR steps’ data in the PL group was: *RPE*_*HIEC*_ *= 0.338 time+ 5.657* (*r*^2^ = 0.93) vs *RPE*_*HIEC*_ *= 0.247 time+ 5.354* (*r*^2^ = 0.98) in the SU group. Slopes, unlike intercepts (*p* = 0.079), were significantly different (*p* = 0.017), suggesting that in the SPR phase, the SU group showed a lower RPE. The linear regression equation of REC steps’ data in the PL group was: *RPE*_*HIEC*_ *= 0.246 time+ 5.513* (*r*^2^ = 0.92) vs *RPE*_*HIEC*_ *= 0.221 time+ 4.452* (*r*^2^ = 0.97) in the SU group. Slopes were not significantly different (*p* = 0.371), while an extremely significant difference was found between intercepts (*p* < 0.001). This implies that in the REC steps, the SU group showed a systematically lower RPE (Fig. 3d and f).

On the whole, RPE values increased linearly over the execution time of HIEC in both the SU and PL groups (Fig. 3a and b). Notably, the extent of the increment was significantly lower in the SU group than it was in the PL group in all the conditions tested (Fig. 3d, e and f), with the only exception of the 1d pre-training SPR phase (Fig. 3c); the lowest increment was observed in the 9w post-training REC phase.

**Fig. 4:**
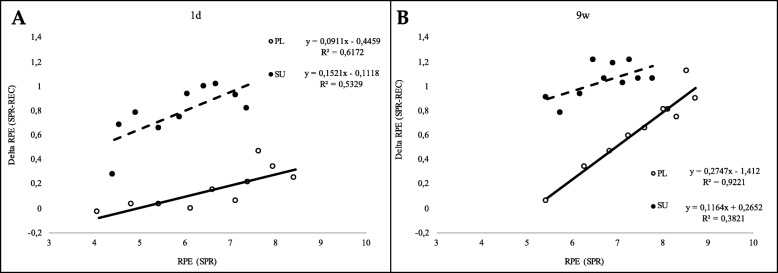
RPE reduction after the completion of each REC step in the SU and PL groups. The RPE differences are expressed as Delta RPE, which represent the difference between the RPE measured at the end of each SPR and at the end of its subsequent REC step. Delta RPE are plotted against the absolute RPE (on the x-axis) measured at the end of each corresponding SPR step. Panel **a** and **b** show 1d and 9 w, respectively

That SU group experienced a more efficient recovery than the PL group,. a lower REC-associated RPE, which can be better appreciated in the scatter plots of Fig. 4a and b showing the differences (Delta) between the SPR- and REC- RPE values as a function of the RPE recorded at the end of each of the SPR steps.

**Fig. 5:**
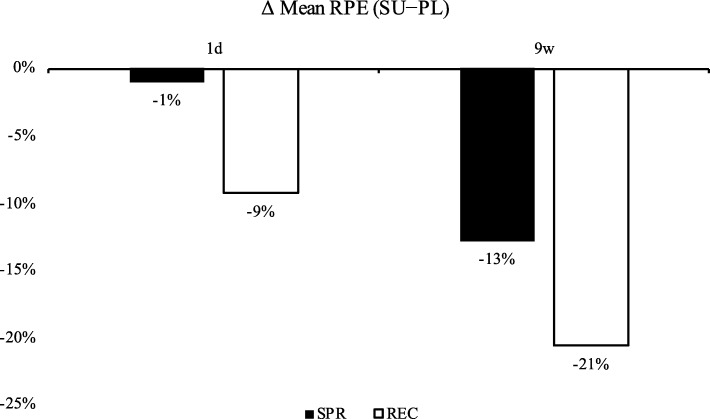
Difference between the mean RPE scores of SU vs PL groups. Solid bars express the percent RPE differences between the SU and PL groups in the SPR phases and open bars, the percent RPE differences in the REC phases; RPE were measured during the 1d (left) and 9w (right) HIEC sessions. The 1d SPR column was calculated from the data points in Fig. 3c; 9w SPR, 1d REC and 9w REC columns were calculated from the data points in Fig. 3d, e and f, respectively

Furthermore, after 9w, the means of the RPE scores in the SU group were reduced compared to the PL group by 13% in the SPR and by 21% in the REC phases; notably, even after the first administration of FP at 1d, RPE during the REC phase decreased by 9% compared to the PL group (Fig. 5).

### Performance during HIEC test: time to exhaustion

TTE values were determined and taken as reliable performance parameters [50, 51]. Analysis of the 1d data failed to reveal significant differences between groups (371 ± 147 s for SU; 359 ± 177 s for PL; *p* > 0.05). On the contrary, with regard to 9w, data showed that the mean TTE was significantly longer for the SU group (517 ± 210 s) than for the PL group (321 ± 214 s) (*p* = 0.025), with partial η^2^ = 0.201 (medium effect); the interaction effect was also significant (*p* < 0.05).

### Training load analysis

**Fig. 6:**
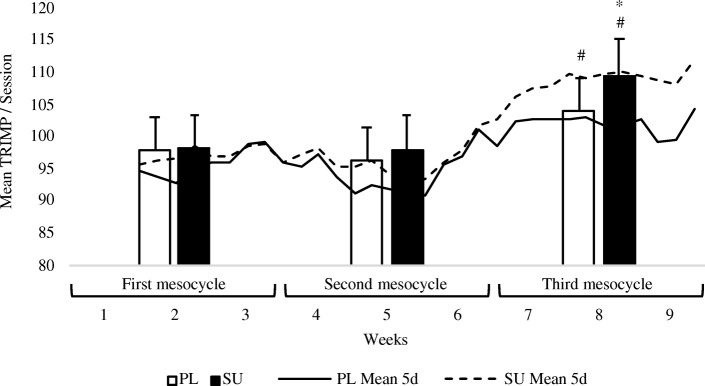
Training loads in the PL and SU groups as a function of mesocycles and training progression. Bars represent the mean training impulse (TRIMP) associated with the corresponding mesocycle in the PL (white columns) and SU (black columns) groups (standard deviations are reported). Mesocycles and weeks are reported on the x axis. Dashed lines (SU) and solid lines (PL) were obtained using a 5-day moving average. * *p* < 0.05 as compared to PL; # *p* < 0.05 as compared to an earlier time point

TRIMP represents a recognized parameter to express the extent of training load [52]. TRIMP values were compared between groups in the course of the training period, which was divided into three different three-week mesocycles (first mesocycle: 1–3 weeks; second: 4–6 weeks; third: 7–9 weeks) characterized by progressively increasing training loads (both in terms of the frequency and duration of the sessions). During the first mesocycle (3 sessions/week of 53.1 ± 1.3 min) subjects averaged 98.4 ± 4.9 TRIMP (SU) and 97.9 ± 4.1 (PL) per session (total TRIMP per mesocycle: 886 in SU, 881 in PL); during the second mesocycle (4 sessions/week, of 59.1 ± 1.2 min), subjects averaged 97.9 ± 5.4 TRIMP (SU) and 96.5 ± 7.1 (PL) per session (total TRIMP per mesocycle: 1175 in SU, 1158 in PL); no differences in these mesocycles were found between groups (post-hoc LSD test; *p* > 0.05). Notably, during the last mesocycle (5 sessions/week of 68.2 ± 1.4 min) TRIMP values were significantly higher (post-hoc LSD test; *p* = 0.014; ES = 0.6, large effect) in the SU group than they were in the PL group, with averages of 109.4 ± 5.7 vs 104.1 ± 6.4 per session, respectively (total TRIMP per mesocycle: 1641 in SU, 1561 in PL). Data are shown in Fig. 6.

### Serum Creatine kinase (CK)

**Fig. 7:**
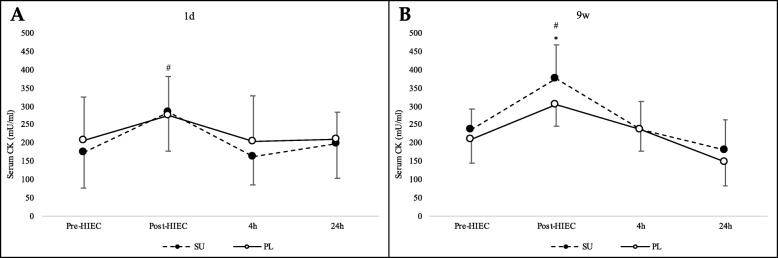
Creatine kinase (CK) serum blood levels. CK was determined at the indicated time points at 1d (**a**) and 9w (**b**) in the SU (black dots) and PL groups (white dots); * *p* < 0.05 as compared to PL; # *p* < 0.05 as compared to an earlier time point

Serum blood CK levels changed over time in the SU and PL groups at both 1d and 9w measurements (*p* < 0.001). At 1d, CK levels showed an increase in the post-HIEC, followed by a progressive decrease before returning to basal values after 24 h. At 1d, group partnership (SU or PL) did not show a different trend of CK concentration (time x group interaction; *p* = 0.568). On the contrary, at 9w SU vs PL group showed a different trend of CK concentration (time x group interaction; *p* = 0.017). A contrast analysis for determining differences between two consecutive measures showed that the SU group was different from the PL group in “post-HIEC *vs* pre-HIEC (*p*=0.048)” and “4h *vs* post-HIEC (*p*<0.047)”. In other words, CK levels were significantly higher only in the SU group in the post-HIEC at 9w, while in all the other conditions, no significant differences could be identified. However, after 4 h, the SU group [CK] was no longer significantly different (*p* > 0.05) from the PL group. Data are shown in Fig. 7.

### Serum blood levels of BCAA, Ala, Trp, Ammonia and ratios of free Trp:BCAA

**Fig. 8:**
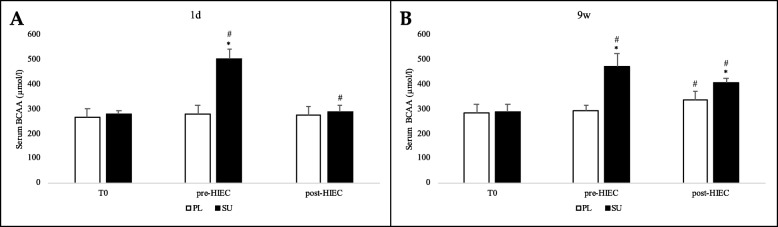
Branched chain amino acids [BCAA] serum blood levels. [BCAA] (total amount of Leu, Isoleu and Val concentrations) were determined prior to (T0) FP or PL powder ingestion, 1 h after (pre-HIEC) and at the end of the HIEC test (post-HIEC). Panels **a** and **b** show analyses performed at 1d and 9w respectively. Values for the SU (black bars) and PL (white bars) groups are reported, with mean and standard deviations. * *p* < 0.05 per group; # *p* < 0.05 per time

Blood samples were collected immediately before (T0), 1 h after the ingestion (pre-HIEC) of FP or PL, and at the end of the HIEC test (post-HIEC). HPLC analysis of serum blood samples (Fig. 8) showed that total BCAA concentrations ([BCAA]) before the ingestion of FP or PL powder at both 1d and 9w were similar, and that at pre-HIEC they increased significantly only in the SU group (*p* < 0.05). [BCAA] measured at post-HIEC decreased significantly in the SU group at 1d and 9w, though to a lesser extent in the latter case.

**Fig. 9:**
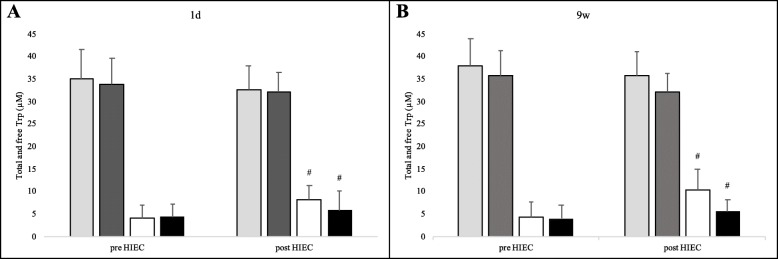
Total and free Trp plasma concentrations. Trp levels were determined at pre-HIEC and at post-HIEC. Panels **a** and **b** show analyses performed at 1d and 9w, respectively. Key: pale grey bars show total Trp in the PL group; dark grey bars, the total Trp in the SU group; white bars, the free Trp in the PL group; black bars, the free Trp in the SU group. Data are reported as means ± standard deviation. # *p* < 0.05 per time

Pre- and post-HIEC plasma levels of total Trp and free Trp were also determined and are shown in Fig. 9: no significant difference (*p* > 0.05) was found in the total Trp values both as a time- or group- function; free Trp levels increased significantly in post-HIEC compared to pre-HIEC, both at 1d (*p* = 0.001) and at 9w (*p* = 0.003), while no significant change was detected between groups (*p* > 0.05).

**Fig. 10:**
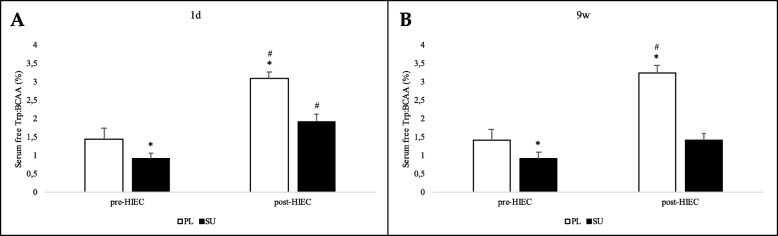
Free Trp to BCAA ratios. Free Trp and BCAA levels were determined and their ratios were then calculated in both the FP and PL groups. Trp:BCAA ratios before (pre-HIEC) and after HIEC test (post-HIEC) are shown. Panel **a** shows analyses performed at 1d and panel **b** those performed at 9w. Values for the SU (black bars) and PL (white bars) groups are reported as means with standard deviations. * *p* < 0.05 between groups; # *p* < 0.05 between time points

Regarding Trp:BCAA ratios, at pre-HIEC they were consistently higher in the PL group than they were in the SU group (Fig. 10). At 1d, notwithstanding the time-related increase in both groups (pre- vs post-HIEC), the PL group was characterized by a higher ratio than the SU group; interestingly, at 9w a statistically significant increase could be found only in the PL group.

**Fig. 11:**
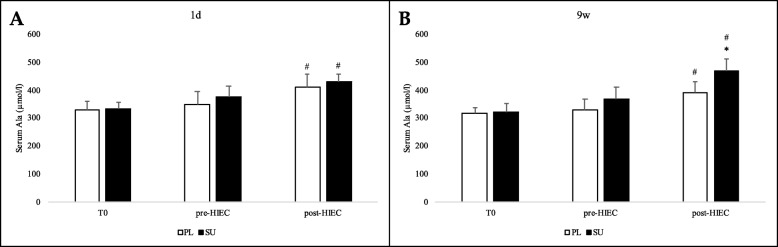
Ala serum blood levels after SU or PL ingestion and post-HIEC. **a** and **b** refer to the beginning (1d) or the end (9w) of the training period, respectively. Values for the SU (black bars) and PL (white bars) groups are reported as means with standard deviations. * *p* < 0.05 between groups; # *p* < 0.05 compared to an earlier time point

Ala serum blood levels ([Ala]) reached slightly higher levels only in the SU group at 1d and 9w pre-HIEC phase (*p* = 0.06; Fig. 11), while in post-HIEC at both time points [Ala] significantly increased in the PL as well as in the SU group (*p* < 0.05), with the latter characterized by a slightly higher increment at 9w vs PL.

Finally, serum ammonia levels at 1d were 40.4 ± 18.0 μM SU vs 43.6 ± 23.2 μM PL at T0; 49.1 ± 22.1 SU vs 42.4 ± 20.3 PL at pre-HIEC; 121.0 ± 78.6 SU vs 111.3 ± 61.2 PL at post-HIEC. At 9w similar values were observed with T0 levels of 43.6 ± 21.5 μM SU and 43.3 ± 24.5 μM PL; 49.3 ± 20.6 SU vs 42.0 ± 20.4 PL at pre-HIEC; 121.1 ± 67.5 SU vs 108.7 ± 51.6 PL at post-HIEC. Statistically significant differences were found only in pre-HIEC vs post-HIEC (*p* < 0.05).

### Glycemia

Glycemia was determined prior to breakfast (4.8 ± 0.1 and 5.3 ± 0.2 mM in SU vs PL respectively, *p* > 0.05) and at different time points up to the end of HIEC test. As expected, 30 min after breakfast, glucose levels increased (9.4 ± 1.5 and 8.5 ± 1.8 mM in the SU and PL groups respectively) and decreased thereafter, approaching basal levels (5.7 ± 0.5 in SU vs 5.6 ± 0.6 mM in PL; *p* > 0.05). No further significant difference between groups was observed post-HIEC (6.1 ± 0.2 vs 5.8 ± 0.6 mM in SU and PL respectively; *p* > 0.05).

## Discussion

The effects of FP -an established, commercially available sports nutritional supplement containing BCAA, Ala and CHO - on RPE, performance and the capacity to sustain physical training were investigated in a group of 32 healthy young subjects enrolled in a randomized double-blind placebo-controlled trial. Along with RPE and performance values, a number of relevant nutritional and biological parameters were also determined. Notably, to the best of our knowledge, this is the first study adopting a validated and reliable HIEC protocol [26] for these purposes. Indeed, other protocols have been used to determine similar end-points in the past [20, 53], but it is worth noting that they had not been previously and specifically validated.

The major finding of this study is that a single intake of FP is capable of attenuating RPE, and that its prolonged 9w consumption according to manufacturer’s recommendations not only augments RPE-attenuating capacity, but also improves TTE and TRIMP, which both reflect the capacity to sustain training loads. HPLC analysis of blood sampled 1 h after FP ingestion, unlike the sample taken 1 h after PL administration, showed a significant increase in BCAA levels. This finding indicates that BCAA are rapidly absorbed after oral ingestion of FP, and that their increased serum blood concentration is likely related to the above-mentioned effects on RPE, TTE and TRIMP.

Following the first intake, the SU group showed lower RPE values only in the HIEC REC phases, while a significant RPE reduction was found following a chronic (9w) intake also in the high intensity SPR phases. Furthermore, both acute and chronic intake caused a significantly more rapid decrease in RPE observed between the SPR and corresponding REC phases compared to PL. It is worth noting that, unlike previous studies on BCAA and RPE [14, 54], by virtue of the particular design of the HIEC test, this is the first investigation in which RPE associated with SPR or with REC phases was separately quantitated. This allowed us to determine that FP significantly accelerated the reduction of RPE during the recovery phases compared to PL.

As regards Trp levels, we only found a slight although significant exercise-dependent variation in free-Trp between pre- and post-HIEC, an effect in line with the data reported and discussed by other Authors [55, 56].

Our results indicate that serum blood circulating Trp:BCAA ratios increase after HIEC in PL, and that FP consumption invariably prevented this effect. Similar qualitative and quantitative results have been observed in previous studies [14, 30] on BCAA supplementation and RPE in exercising young adults. Under the conditions we observed in the PL group, namely an increased Trp:BCAA ratio, Trp is supposed to be more available for brain uptake, thus promoting an augmented synthesis of serotonin [23]; on the contrary, a significantly lower Trp:BCAA ratio, which we did observe in the SU group, is thought to antagonize brain Trp uptake, thus limiting serotonin synthesis and availability [57]. According to the widely held belief linking brain serotonin increases with central fatigue development [7, 14], this sequence of events might have contributed to the lower RPE values we observed upon acute and/or prolonged FP supplementation. Since in our conditions Trp blood levels increase, some concern might be raised with regard to its conversion, through the kynurenine pathway, into correspondingly higher levels of the excitotoxic quinolinic acid and kynurenine [58]. However, as discussed by Fernestrom et al. [59], even under conditions of supplementation with extra-Trp, no effect attributable to quinolinic acid toxicity have never been observed in humans. In addition, physical exercise has been shown to prevent per se the eventual brain entry of Trp-derived kynurenine [60] as well as to attenuate the activity of the kynurenine pathway [61, 62].

Ammonia cerebral uptake and concentration are known to increase in humans during prolonged exercise [12], thus augmenting central fatigue by altering cerebral energy metabolism and neurotransmission [8]. However, although HIEC promotes an increase in serum ammonia levels, we did not find differences between the SU and PL groups at any of the considered time points (T0, pre-HIEC and post-HIEC). This finding, in keeping with data from the literature [15], might depend on the relatively low dose of supplemented BCAA.

With regard to the higher [Ala] upon FP ingestion, we can only speculate on its relevance based on the literature. Supplemental Ala has been shown to exert a positive influence on the anaplerosis of the tricarboxylic acid cycle, on muscle glycogen storage, energy synthesis and on the regulation of ammonia metabolism, transport and excretion [63, 64]. Along these same lines, although we have no direct evidence, higher [Ala] could exert a converging role in support of the effects on RPE observed herein.

Regarding glycemia, we did not find any variation between the two groups in the glycemic values of pre- and post-HIEC tests, suggesting that the extra CHO of FP do not significantly modify blood glucose prior to or after testing compared to PL. In this regard, it should also be considered that in our setting both groups had ingested a breakfast containing 120–150 g of CHO 1 h before HIEC, that is approximately tenfold the amount of CHO contained in FP. In light of these considerations, the CHO contribution to the functional and metabolic outcomes described thus far is probably limited. Indeed, a recent study by O’Hara et al. [65], using the same experimental setting we adopted in the present investigation, showed that the intake of 40 g of CHO (galactose or glucose) in one liter of water, taken 30 min before HIEC, did not modify the RPE or the TTE compared to the placebo.

Finally, with respect to the possible direct effects of CHO on RPE, only in studies in which CHO were given during -and not prior to (as in our case) - endurance exercise have such effects been observed [66]. On the whole, it can be inferred that in our conditions CHO hardly affect RPE through direct central interactions.

With regard to performance, most of the studies on BCAA-containing supplements have failed to find any significant improvements [54, 67] nor did we find any differences in terms of relevant metabolic parameters (VO_2max_ and Power at Lactate Thresholds) between SU and PL, either upon single (1d) or prolonged (9w) supplementation. However, even though TTE did not improve after the first, acute intake of FP, it did increase significantly following the 9w supplementation. This observation is in line with those of Kephart et al. [22], showing that, although in a different experimental settings, 10-week BCAA supplementation results in increased peak/mean power in well-trained cyclists. Interestingly, the same study also reported a significant increase in serum blood [BCAA] and a consequent improvement in the circulating Trp:BCAA ratio, hence suggesting that performance enhancement could be related to a central fatigue-mediated mechanism [22]. Considering that our SU group did not show any improvement in metabolic parameters or free-fat mass (not shown), we also suggest that the TTE increase might be related to the stable attenuation of RPE rather than to ergogenic or anabolic effects.

With regard to the ability to sustain training loads, our results showed that TRIMP were the same in both groups with work volumes per week < 240 min. Interestingly, at higher work volumes (ca. 350 min in the third mesocycle) TRIMP values were significantly higher in the SU than in the PL group. In this regard, it is worth considering that higher TRIMP expresses an increased ability to sustain exercise at high HR values, while lower TRIMP reflects the relative inability to exercise under the same conditions.

Several studies report that the inability of athletes to increase their HR for a given load is indicative of an overreaching state [68, 69]. Again, in accordance with the serotonin theory of central fatigue, chronic elevation in brain serotonin levels has been causally associated with the development of an overtraining state and related symptoms, culminating in decreased performance [70]. Although it is mere speculation, the improved Trp:BCAA ratios afforded by FP supplementation could also explain the enhanced capacity to sustain higher training loads in SU athletes.

Elevation of serum blood CK within 24/72 h post-exercise is recognized as a marker of muscle damage caused by intense eccentric and resistance training [71, 72], and its severity also depends on exercise intensity [73]. BCAA supplementation, under specific circumstances (high dosage,. 12–20 g/day for at least 10 days starting 1 week before challenging exercise) has been shown to prevent the elevation of serum CK levels following a continuous, submaximal exercise test, thus suggesting that it may attenuate muscle damage [21, 74]. Our testing conditions also involved 10 sprints and a TTE phase performed at 90% of W_peak_, and could reasonably result in some muscle damage. However, despite the exhaustive protocol adopted, we did not find serum CK variations ascribable to muscle damage. Indeed, CK level increases were transient and returned rapidly (4 h) to baseline values, showing no variations thereafter (24 h) in in either the SU or PL group. On the other hand, we found that after 9w of supplementation, the transient post-HIEC increase in CK was significantly higher in the SU group than it was in the PL group, an effect that could be accounted for by the higher training load of the SU group. In spite of this more consistent serum CK increase, 4 h after completion of HIEC, the SU group recovered to the same baseline values as the PL group. These results suggest that the transient CK increase in our conditions is not indicative of muscle damage, but is rather an expression of the higher training load [75].

On the whole, our data suggest that the higher TRIMP values found in SU subjects at 9w reflect their enhanced capacity to sustain training, whose volume may consequently increase over time leading to better performance than that achieved by PL subjects. Reduction in RPE, which was observed from the very beginning of the test period, is likely to play a pivotal role in the progressively enhanced capacity to sustain higher training volumes. The main limitation of the present study, as well as of similar ones, lies in the use of a multi-ingredient supplement, which makes it difficult to determine the relative impact of each component on the tested markers: as a consequence, ascertaining which of the ingredients had what effect or if there was a synergistic interaction among the ingredients remains an open question. On the other hand, the strength of this study resides in the fact that it details a multi-technique experimental approach that could be applied, in the future, to directly compare the efficacy of formulations containing different constituents (such as caffeine, electrolytes, β-alanine etc.) in attenuating RPE. This would be important because, at present, it is very hard to compare the effects of different sport supplements with different formulations on RPE because they have been studied using non-homogeneous experimental designs and approaches [76].

## Conclusions

The main findings of this study are that the consumption of FP (a commercially available nutritional supplement containing BCAA, Ala and CHO) according to the producer’s suggestions reduces RPE at all the time points tested and that, over a 9w-intake, also improves TTE and TRIMP. Although it was not possible to specifically address mechanistic issues, the effects we observed are in keeping with the theory of RPE sensitivity to serum blood Trp:BCAA ratio, while the contribution of metabolic effects seems negligible. The prolonged intake of FP, which promotes a reduction in RPE and recovery times, can enhance the capacity to sustain higher training loads and ultimately improve endurance performance. Importantly, these effects occur without affecting dietary habits and caloric intake.

## Data Availability

The datasets used and/or analyzed during the current study are available from the corresponding author upon reasonable request.

## Abbreviations

*1d*: 1 day
*9w*: 9 weeks
Ala: Alanine
BCAA: Branched-Chain Amino Acids
CHO: Carbohydrate
CK: Creatine Kinase
CNS: Central Nervous System
FP: Friliver Performance
HIEC: High Intensity Endurance Cycling
HIIT: High Intensity Interval Training
HR: Heart Rate
LT: Lactate threshold
PL: Placebo group
RDA: Recommended Dietary Allowance
REC: Recovery phase
RPE: Rating of Perceived Exertion
SPR: Sprint phase
SU: Supplemented group
TRIMP: Training Impulse
Trp: Tryptophan
TTE: Time To Exhaustion
VO_2max_: Maximal oxygen consumption
W: Watt
W_LT_: Power at lactate threshold
W_peak_: Peak power
